# A manganese photosensitive tricarbonyl molecule [Mn(CO)_3_(tpa-κ^3^*N*)]Br enhances antibiotic efficacy in a multi-drug-resistant *Escherichia coli*

**DOI:** 10.1099/mic.0.000526

**Published:** 2017-09-28

**Authors:** Namrata Rana, Helen E. Jesse, Mariana Tinajero-Trejo, Jonathan A. Butler, John D. Tarlit, Milena L. von und zur Muhlen, Christoph Nagel, Ulrich Schatzschneider, Robert K. Poole

**Affiliations:** ^1^​ Department of Molecular Biology and Biotechnology, The University of Sheffield, Sheffield, UK; ^2^​ Institut für Anorganische Chemie, Julius-Maximilians-Universität Würzburg, Würzburg, Germany; ^†^​ Present address: Cell Biology Program, The Hospital for Sick Children, Toronto, Canada.; ^‡^​ Present address: School of Healthcare Science, Manchester Metropolitan University, UK.

**Keywords:** antibiotic resistance, antimicrobial agents, bacterial pathogenesis, carbon monoxide, *Escherichia coli*, manganese complex, photosensitive tricarbonyl complex

## Abstract

Carbon monoxide-releasing molecules (CORMs) are a promising class of new antimicrobials, with multiple modes of action that are distinct from those of standard antibiotics. The relentless increase in antimicrobial resistance, exacerbated by a lack of new antibiotics, necessitates a better understanding of how such novel agents act and might be used synergistically with established antibiotics. This work aimed to understand the mechanism(s) underlying synergy between a manganese-based photoactivated carbon monoxide-releasing molecule (PhotoCORM), [Mn(CO)_3_(tpa-κ^3^*N*)]Br [tpa=tris(2-pyridylmethyl)amine], and various classes of antibiotics in their activities towards *Escherichia coli* EC958, a multi-drug-resistant uropathogen. The title compound acts synergistically with polymyxins [polymyxin B and colistin (polymyxin E)] by damaging the bacterial cytoplasmic membrane. [Mn(CO)_3_(tpa-κ^3^*N*)]Br also potentiates the action of doxycycline, resulting in reduced expression of *tetA,* which encodes a tetracycline efflux pump. We show that, like tetracyclines, the breakdown products of [Mn(CO)_3_(tpa-κ^3^*N*)]Br activation chelate iron and trigger an iron starvation response, which we propose to be a further basis for the synergies observed. Conversely, media supplemented with excess iron abrogated the inhibition of growth by doxycycline and the title compound. In conclusion, multiple factors contribute to the ability of this PhotoCORM to increase the efficacy of antibiotics in the polymyxin and tetracycline families. We propose that light-activated carbon monoxide release is not the sole basis of the antimicrobial activities of [Mn(CO)_3_(tpa-κ^3^*N*)]Br.

## Introduction

Carbon monoxide-releasing molecules (CORMs) were originally developed to deliver carbon monoxide (CO) safely in experimental and therapeutic settings [1], in recognition of mounting evidence that CO is an important signalling molecule [2] with opportunities for therapeutic exploitation [3]. A wide range of CORMs has now been developed and diverse modifications have been employed to control and manipulate the CO release triggers and kinetics [4–7].

It is axiomatic that CO is a competitive inhibitor of O_2_ binding to ferrous haem proteins, especially respiratory terminal oxidases and globins. At high ratios of CO : O_2_ (typically ~9 : 1), CO inhibits aerobic respiration [8]. It was therefore predicted that CORMs might possess antimicrobial activity and that these actions would be primarily due to respiratory inhibition [9, 10]. Although CORM-2 and CORM-3 do inhibit aerobic respiration of *E. coli* [10–12], *P. aeruginosa* [13, 14] and other bacteria, mounting evidence is highlighting the multifaceted effects of CORMs, which cannot solely be due to CO [15, 16]. Firstly, CORM-2 and CORM-3 are more potent inhibitors of growth and/or aerobic respiration than CO gas itself, implying that Ru(II) or some other decomposition products of the CORM enhance inhibition [10, 14]. Secondly, transcriptomic studies revealed diverse changes in gene expression, not limited to respiratory function, especially involving membrane stress and drug efflux systems in response to CORM-2 and CORM-3 but not CO gas [10, 17, 18]. Thirdly, despite the fact that the classical targets of CO are ferrous haem proteins, CORM-3 is toxic to haem-deficient *E. coli* mutants and the naturally haem-deficient Gram-positive bacterium *Lactococcus lactis* [19].

The antimicrobial potency of CORMs is poorly understood, a problem confounded by the potential toxicity of the ruthenium (Ru) in the most commonly used CORMs [18, 19]; this led us to explore a manganese (Mn)-based photoactivated carbon monoxide-releasing molecule (PhotoCORM) [Mn(CO)_3_(tpa-κ^3^*N*)]Br [20]. The term PhotoCORM [21] describes a compound that releases CO when it is exposed to light [22]. Such compounds have the advantage that CO release can be precisely controlled, and comparisons of the biological effects of the pro-drug versus the CO-depleted or photo-activated compound allow us to discern the role of CO and that of the metal-coligand fragment. Moreover, many advances have been made recently in the field of photoactivated chemotherapy (PACT) [23], including in the treatment of cancer [24], thus providing the technology required to make the therapeutic use of PhotoCORMs a possibility. [Mn(CO)_3_(tpa-κ^3^*N*)]Br, upon photoactivation, inhibits the growth and reduces the viability of *E. coli* K-12 [20] and the uropathogenic *E. coli* (UPEC) EC958 [25]. The mechanism of toxicity of this compound is not fully understood, but CO released from the PhotoCORM binds to respiratory cytochromes and inhibits respiration, while metal ion acquisition is perturbed.

This work focuses on *E. coli* EC958, a clinical isolate of *E. coli* ST131 [26]. The multidrug-resistant UPEC clone belonging to serotype O25b:H4 and sequence type *E. coli* ST131 has emerged as a major cause of urinary tract and bloodstream infections, and a worldwide public health threat [27]. *E. coli* ST131 produces cefotaxime (CTX)-M-type extended spectrum β-lactamases (ESBLs) [28] and exhibits resistance to β-lactams and several classes of non-β-lactams (e.g. fluoroquinolones), significantly limiting treatment options [29, 30]. The increasing prevalence of ST131 strains requires novel antimicrobial strategies to combat infections.

One approach to combat antibiotic resistance is the use of combinatorial therapies with the aim of reducing dependence on single antibiotics [31]. Previously, we showed that the title compound potentiates the effects of the antibiotic, doxycycline (DOX), which binds to the 30S subunit of the bacterial ribosome, preventing protein synthesis [25]. Here we set out to investigate whether [Mn(CO)_3_(tpa-κ^3^*N*)]Br is synergistic with other antibiotics against strain EC958, specifically those that: inhibit protein synthesis at the 30S subunit of the bacterial ribosome [gentamycin (GEN), kanamycin (KAN) and tetracycline (TET)]; bind to the 50S subunit [chloramphenicol (CHL)]; interact with penicillin-binding proteins, thus inhibiting cell wall biosynthesis [cefotaxime (CTX)]; inhibit dihydrofolate reductase activity and DNA synthesis [trimethoprim (TMP)]; or bind to and disrupt the outer membrane [colistin (CST) and polymyxin B (PMB)]. We investigate the basis of the synergy and show that iron starvation and membrane damage are key to understanding the mechanism of toxicity of the PhotoCORM, both alone and in the presence of antibiotics.

## Methods

### PhotoCORM preparation and use

The synthesis of [Mn(CO)_3_(tpa-κ^3^*N*)]Br was described previously [20]. Aqueous stock solutions (10 mM) were kept in the dark for up to 24 h at 4 °C. The PhotoCORM was activated by adding PhotoCORM to cells and immediately illuminating the culture for 6 min at 365 nm with a UV lamp (UVITEC, Cambridge) placed 3 cm above the sample. CO-depleted PhotoCORM was prepared by exposing 1 ml of PhotoCORM (3 mM) to UV light at 365 nm for 30 min with stirring.

### Determination of minimum inhibitory concentrations (MICs) using E-tests

E-tests were performed in accordance with the manufacturer’s instructions (bioMérieux SA). Briefly, inocula of *E. coli* EC958 and MG1655 were prepared and adjusted to 0.5 McFarland standard and spread onto Mueller*–*Hinton II agar plates. The MIC breakpoints for defining interpretive categories published by EUCAST [32] were used for interpreting E-test MIC values.

### Growth conditions

Starter cultures of EC958 were grown in LB (Luria-Bertani) broth overnight at 37 °C, 200 r.p.m. After centrifugation, cells were suspended in minimal medium [33] or Fe-supplemented minimal medium [in which the basal FeCl_3_ concentration (31 µM) was increased by 10- or 50-fold] with glucose (20 mM) as the sole carbon source. This suspension was used to inoculate fresh medium [10^6^ colony-forming units (c.f.u.) ml^−1^] in 96-well plates and the cultures were treated with [Mn(CO)_3_(tpa-κ^3^*N*)]Br and/or various antibiotics (polymyxins, tetracyclines and aminoglycosides) as described in the Results section. For studies with iron chelators, cultures were treated with citric acid, desferrioxamine or 8-hydroxyquinoline in the presence or absence of the PhotoCORM. To activate the PhotoCORM, 96-well plates with 200 µl culture volumes were exposed to UV as above. Plates were incubated overnight at 37 °C, 200 rpm, in a Sunrise microplate reader (TECAN).

### Checkerboard experiments

Experiments were performed in 96-well plates as described before [31, 34]. Cultures were incubated at 37 °C with shaking for 24 h using a Tecan Sunrise plate reader [See Fig. S1 (available in the online Supplementary Material) for more information]. The concentrations of antibiotics (Sigma) tested were up to four eightfold dilutions lower than the MIC and, where possible, two twofold dilutions higher than the MIC. The MIC was considered as the lowest concentration of the agent alone, or combined with CORM-401, that inhibited growth. The fractional inhibitory concentration index (FICI) [31] was calculated to determine drug interaction, and interpreted as follows: FICI of two-drug combination=FIC_A_+FIC_B_, where FIC_A_ is the MIC of drug A in combination with CORM-401/MIC of drug A alone and FIC_B_ is the MIC of drug B in combination with CORM-401/MIC of drug B alone. The results indicate synergy when the calculated FICI ≤0.5, no interaction when FICI >0.5–4 and antagonism when the FICI >4 [35].

### Metal uptake analyses

Aerobic cultures of EC958 were grown to mid-exponential phase (OD_600_~0.4). To measure intracellular Mn, cultures were treated with either 50 µM or 150 µM activated PhotoCORM alone or in combination with the following antibiotics: CST (0.5 µg ml^−1^); DOX (12.5 µg ml^−1^); OTC (12.5 µg ml^−1^). These lower concentrations of PhotoCORM were used in an effort to minimize metabolic inhibition, since we were interested in determining whether the CORM, assayed as Mn, was actively transported, whereas to measure intracellular Fe, cultures were treated with 300 µM activated PhotoCORM to test for iron depletion from the medium by the PhotoCORM. Cultures were incubated for 40 min before samples were taken and analysed by inductively coupled plasma mass spectrometry (ICP-MS) as previously described [10] using values from the literature for single-cell dry mass and volume [36]. Intracellular metal ion concentrations were determined for cell pellets; extracellular concentrations were determined for supernatant and wash fractions.

### Liposome leakage assay

Liposomes were prepared as described previously [37]. Briefly, liposomes consisting of only (1,2-dioleoyl-*sn*-glycero-3-phospho-1′-*rac*-glycerol) (sodium salt) (DOPG) (Avanti Polar Lipids) or DOPG in combination with 1,2-dioleoyl-*sn*-glycero-3-phosphoethanolamine (DOPE) in the ratio DOPE:DOPG (7 : 3 mol parts) and containing 50 mM 5(6)-carboxyfluorescein (Thermo Fisher) were prepared by extrusion through 0.2 µm polycarbonate filters (Merck Millipore). Excess dye was removed by filtration through a Sephadex G-25 column using 10 mM HEPES pH 7.4 as the eluting buffer. To measure the rate of 5(6)-carboxyfluorescein leakage, the liposomes were diluted (1/6000) in 10 mM HEPES buffer, pH 7.4. The fluorescence emission at 517 nm (excitation at 495 nm) was monitored with a Varian Cary Eclipse fluorescence spectrophotometer (Agilent Technologies) prior to and following the addition of the PhotoCORM (50 µM). The sample was then exposed to UV (365 nm, 6 min) and fluorescence monitoring was resumed. The membrane-disrupting antibiotic CST (30 µM) was used as a positive control and total leakage was determined by the addition of Triton X-100 (0.01 %) (Sigma).

### Inner-membrane depolarization assay

The cytoplasmic membrane depolarization activity of the PhotoCORM in respect of *E. coli* EC958 was determined as described before [38]. In the present study, CST (3 µM) was used as a positive control to determine the total cytoplasmic membrane depolarization as measured using the membrane potential-sensitive fluorescent dye 3,3′-dipropylthiadicarbocyanine iodide [diSC_3_(5)].

### RT-PCR

Exponential-phase cultures were treated with the PhotoCORM (150 µM) alone or in combination with DOX (9 µg ml^−1^), or CST (1 µg ml^−1^). Following treatment, cultures containing the PhotoCORM were exposed to UV (365 nm, 6 min) and then incubated at 37 °C for 10 min with shaking at 200 r.p.m. RT-PCR experiments were performed as before [25]. The primer sets used were: *katG*, 5′ CCATAACACCACAGCCACTG 3′ and 5′ AGTTGATTTGGCCACCAGTC 3′; *sodA*, 5′ TGAGCTATACCCTGCCATCC 3′ and 5′ TCTGATGGTGTTTGGTGTGG 3′; *ahpC*, 5′ GAAATCACCGAAAAAGATACCG 3′ and 5′ CAGTTCTTCGT AATGGTCAGCA 3′; *recA*, 5′ TCTACCGGTTCGCTTT CACT 3′ and 5′ CGTGGTTTTACCGGAAGATT 3′; *tetA*, 5′ CAGACGTGAAACCCAACAGAC 3′ and 5′ ACGTCGTTCGAGTGAACCAG 3′; and *gyrA*, 5′ GGTACACCGTCGCGTACTTT 3′ and 5′ TACCGATTACGTCACCA ACG 3′.

### Iron chelation assay

Chrome Azurol S (CAS) assay solution was prepared as before [39]. Samples of the PhotoCORM (300 µM or 1.5 mM), the CO-depleted PhotoCORM (300 µM), DOX (9 or 96 µg ml^−1^), tris(2-pyridylmethyl)amine (tpa), manganese(II) perchlorate (Mn(ClO_4_)_2_), or CO gas (gently bubbled through the sample for 5 min) were added alone or in combination to either Tris-HCl buffer (50 mM, pH 7.4) or a suspension of EC958 cells. Samples were kept in the dark or illuminated at 365 nm for 6 min. Samples containing bacteria (OD_600_~0.8) were then centrifuged (5000 r.p.m.) and 500 µl supernatant was incubated at room temperature for 1 h with 500 µl CAS assay solution. A UV visible spectrum was then recorded (300–800 nm) and the absorbance at 630 nm was noted for each sample.

## Results

### Light-activated [Mn(CO)_3_(tpa-*κ*^3^N)]Br potentiates the inhibitory effects of antibiotics

*E. coli* strain EC958 was subjected to E-test strips (Epsilometer tests; bioMérieux) – pre-defined, stable gradients of 15 antimicrobial concentrations on a plastic strip, routinely used to assess antibiotic sensitivity. In contrast to laboratory *E. coli* strain MG1655, EC958 was resistant to multiple classes of antibiotics, including tetracyclines (DOX and TET), an aminoglycoside (KAN) and a dihydrofolate reductase inhibitor (TMP) (Table S1). These findings are predicted by the genome sequence, which shows that plasmid pEC958 includes genes for antibiotic resistance, including *tetA* and *tetR* (encoding tetracycline resistance proteins), *aac6′-lb-cr* [encoding aminoglycoside N(6*′*)-acetyltransferase] and *dhfrVII* (encoding dihydrofolate reductase [26]).

**Fig. 1.**
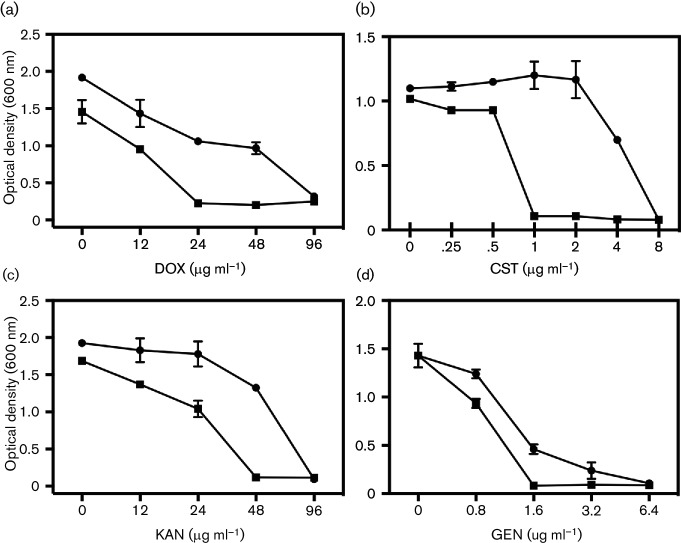
Activated PhotoCORM [Mn(CO)_3_(tpa-κ^3^*N*)]Br increases the efficacy of certain antibiotics. Cultures were treated with increasing concentrations of antibiotics in the absence (⚫) or presence of 200 µM PhotoCORM (■) in minimal media and illuminated for 6 min at 365 nm. The antibiotics were: DOX (a); CST (b); KAN (c); GEN (d). OD_600nm_ was recorded after 24 h aerobic incubation at 37 °C, 200 r.p.m. The graphs show the averages ±sem of three biological repeats. Where error bars appear to be missing, the errors are within the dimensions of the experimental point.

**Fig. 2.**
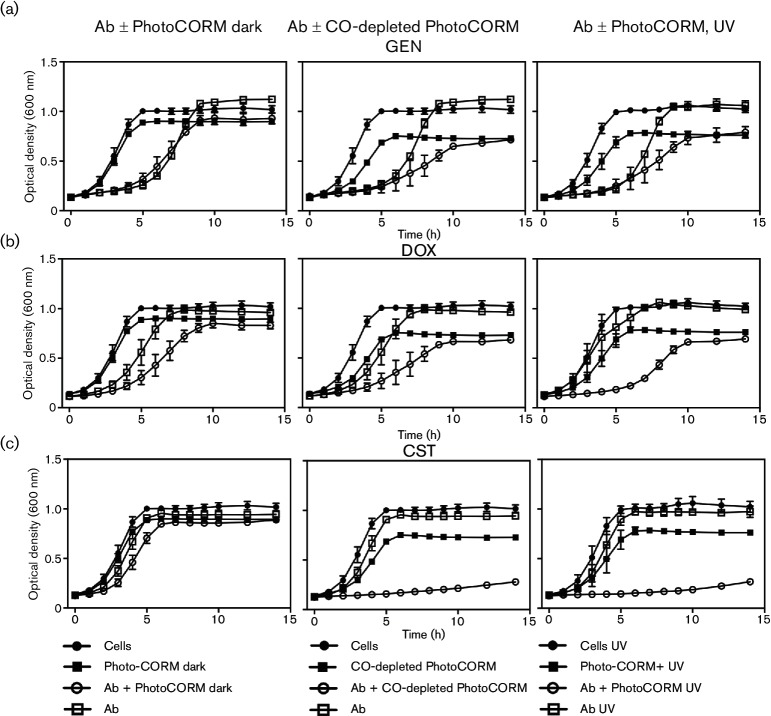
Combining sub-inhibitory concentrations of antibiotics and [Mn(CO)_3_(tpa-κ^3^*N*)]Br increases growth inhibition. Cultures were treated with antibiotics alone [GEN, 0.6 µg ml^−1^ (a); DOX, 9 µg ml^−1^ (b); and CST, 1 µg ml^−1^ (c)] or combined with 200 µM PhotoCORM (either illuminated, dark or CO-depleted). Ab, antibiotic; UV, cultures illuminated at 365 nm for 6 min. Shown are: cells (no additions), (⚫); antibiotic alone (□); PhotoCORM alone (dark, CO-depleted or with UV, ■); and a combination of antibiotic and the PhotoCORM (O). Graphs show the averages ±sem of three biological repeats.

To investigate whether [Mn(CO)_3_(tpa-κ^3^*N*)]Br enhances the inhibitory effects of antibiotics, EC958 cultures were treated with increasing concentrations of antibiotics in the absence or presence of a concentration of the activated PhotoCORM (200 µM) that elicited partial inhibition of growth [25]; cells were treated with the compound and illuminated *in situ* at 365 nm to allow for CO release (Fig. 1). Activated PhotoCORM reduced the MICs of certain antibiotics, including DOX (reduced from 96 to 24 µg ml^−1^), CST (8 to 1 µg ml^−1^), KAN (96 to 48 µg ml^−1^) and GEN (6.4 to 1.6 µg ml^−1^), whereas the MICs for CTX and CHL were unaltered (not shown). UV-activated [Mn(CO)_3_(tpa-κ^3^*N*)]Br (200 µM) also potentiated the growth inhibitory effects of GEN, DOX and CST (Fig. 2a–c, right-hand panels). The effect was most marked with DOX (Fig. 2b) and especially CST (Fig. 2c). However, CO-depleted PhotoCORM, i.e. the PhotoCORM solution illuminated *ex vivo* to allow CO to be released, also enhanced inhibition by GEN and DOX (Fig. 2a, b, middle) and dramatically potentiated the effect of CST (Fig. 2c, middle). Even the non-illuminated PhotoCORM slightly increased growth inhibition by DOX (Fig. 2b). Thus, the released CO is not the main synergistic partner of these antibiotics.

### Nature of the interactions between antibiotics and the PhotoCORM

Checkerboard experiments were conducted, in which increasing concentrations of the title compound were titrated against increasing concentrations of the antibiotic (for details see Fig. S1). A combination was considered to be synergistic when the sum of the fractional inhibitory concentrations (ΣFIC) was ≤0.5, non-interacting when >0.5 and <4, and antagonistic when the ΣFIC was ≥4 [35]. These confirmed that activated PhotoCORM functioned synergistically with antibiotics that target the cell membrane; for CST (ΣFIC=0.38) the MIC was reduced eightfold (from 8 to 1 µg ml^−1^) (Table 1). The data from the checkerboards and ΣFIC calculations are shown in Table S2. Synergy was also seen with the PhotoCORM and PMB (ΣFIC=0.25), where the MIC of PMB was also reduced eightfold, and with PhotoCORM and the human cathelicidin peptide LL37 [40] (ΣFIC=0.5). Synergy was also seen with antibiotic inhibitors of protein synthesis (Table 1). For DOX, the ΣFIC was 0.28 and the activated PhotoCORM reduced the MIC by fourfold. However, no interaction was found with the PhotoCORM and TET (ΣFIC=0.75). OTC was non-inhibitory and gave no useful MIC. These diverse interactions of the activated PhotoCORM with members of the tetracycline family may be explained by the different sensitivities of EC958 to these antibiotics. Among the tetracyclines, DOX had the lowest MIC against EC958 (Table 1), whereas the MICs of TET and OTC were over twofold higher (>240 µg ml^−1^). Thus, a threshold of susceptibility may be required for synergism to occur. To test this hypothesis, checkerboard experiments were also performed with MG1655, an *E. coli* strain that is sensitive to both tetracycline and OTC. The PhotoCORM was synergistic with both these antibiotics against this strain (Table 1).

**Table 1.** Checkerboard analysis of the synergistic effects of the light-activated PhotoCORM with certain antibiotics

Bacterial strain	Antibiotic	MIC_antibiotic_* alone (μg ml^−1^)	MIC_antibiotic_ with PhotoCORM (μg ml^−1^)	Σ FIC	Interaction
EC958	CST	8	1	0.38	Synergy
	DOX	100	25	0.28	Synergy
	LL37	32	8	0.5	Synergy
	OTC	>240	>240		No interaction
	PMB	2	0.25	0.25	Synergy
	TET	>240	60	0.75	No interaction
MG1655	OTC	4	0.25	0.31	Synergy
	TET	2	0.5	0.31	Synergy

*Minimum inhibitory concentration of antibiotic. Fractional inhibitory concentration. Values are representative of ≥3 independent biological repeats.

### Antibiotics do not increase accumulation of Mn from PhotoCORM in EC958 cells

Since CORM-3 is taken up by bacteria [10], we investigated whether the synergistic interactions between [Mn(CO)_3_(tpa-κ^3^*N*)]Br and CST were due to increased PhotoCORM (and therefore Mn) uptake in the presence of antibiotics. Cultures were treated with UV-activated [Mn(CO)_3_(tpa-κ^3^*N*)]Br (50 or 150 µM), alone or in combination with CST (0.5 µg ml^−1^) (Fig. S2). After incubation for 40 min, cell pellets, culture supernatants and wash fractions were analysed for Mn content via ICP-MS. No increased accumulation of Mn was observed when cells were treated with a combination CST and the PhotoCORM (Fig. S2). We previously showed that the CORM does not accumulate measurably in the absence of antibiotics [25].

### The PhotoCORM contributes to bacterial membrane damage

**Fig. 3.**
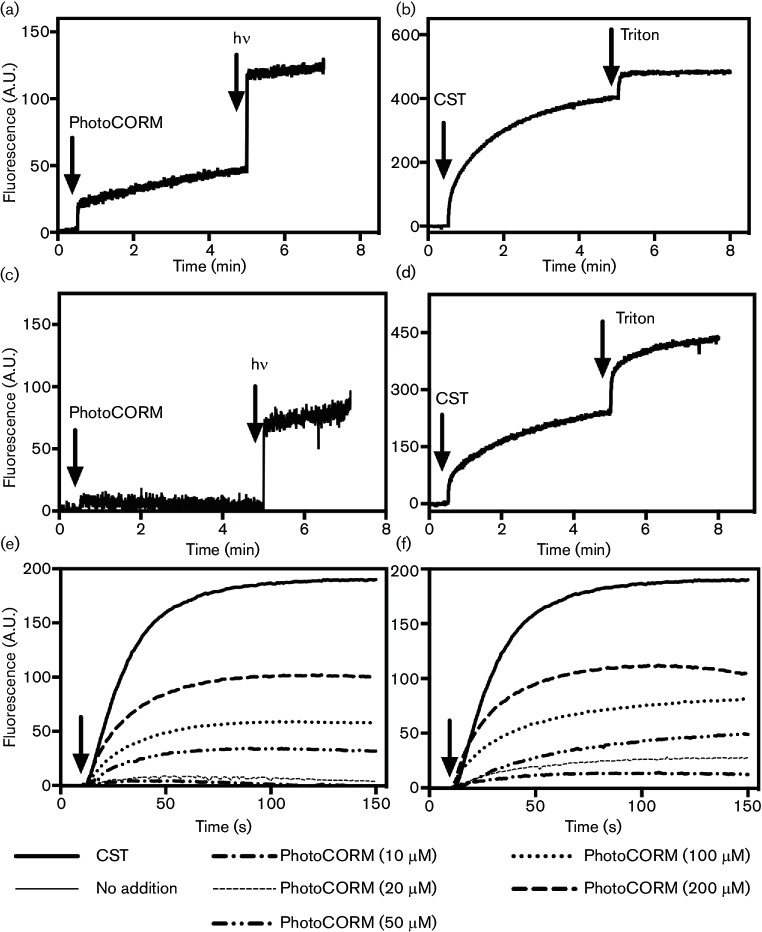
PhotoCORM perturbs membrane integrity. Membrane damage by [Mn(CO)_3_(tpa-κ^3^*N*)]Br assessed by carboxyfluorescein release from liposomes prepared from (a), (b) DOPG only or (c), (d) a DOPE–DOPG mix in a 7 : 3 ratio. (a) Liposomes were treated with PhotoCORM (50 µM) at the first arrow and then illuminated for 6 min (second arrow). (b) A control experiment with liposomes but no PhotoCORM: the first arrow shows the addition of CST (30 µM) and the second arrow shows the addition of 0.01 % Triton. Panel (c) is as (a), except with a 7 : 3 mix of DOPE–DOPG, and (d) is the corresponding no-PhotoCORM control. The excitation wavelength was 495 nm and emission was at 517 nm. (e), (f) Cytoplasmic membrane depolarization of *E. coli* EC958 by [Mn(CO)_3_(tpa-κ^3^*N*)]Br measured using the membrane potential-sensitive dye, diSC_3_(5). (e) Cells treated with the PhotoCORM without illumination at a range of concentrations. (f) The PhotoCORM illuminated prior to addition to cells. CST (10 µM) was used as the positive control. The excitation wavelength was 622 nm and emission was at 670 nm. The data shown are representative of three technical repeats of two independent experiments.

We investigated the ability of [Mn(CO)_3_(tpa-κ^3^*N*)]Br to damage membranes by measuring the release of a fluorescent dye (5(6)-carboxyfluorescein), encapsulated at a self-quenching concentration (50 µM), from liposomes composed of 1,2-dioleoyl-*sn*-glycero-3-phosphoglycerol (DOPG) with an overall negative charge, or from 1,2-dioleoyl-*sn*-glycero-3-phosphoethanolamine (DOPE)– DOPG (7 : 3 ratio), which has a more neutral charge (Fig. 3a–d). In Fig. 3a, PhotoCORM (50 µM) was added to liposomes at 0.5 min (first arrow) and the changes in fluorescence were recorded. The sample was then illuminated (second arrow) and further changes in fluorescence were measured. Increased fluorescence indicated that the PhotoCORM elicits dye leakage from liposomes and that the extent of leakage increased on illumination. Total membrane damage was determined by the addition of CST (10 µM) and Triton X-100 (0.01 %), which resulted in maximum leakage (Fig. 3b, d). The PhotoCORM caused damage to DOPG (i.e. negatively charged) liposomes, both before and after illumination, but only damaged the more neutral DOPE–DOPG liposomes after illumination (Fig. 3c, second arrow). This suggests that negatively charged DOPG liposomes are more susceptible to damage by the non-activated PhotoCORM. The membrane charge may be an important factor since, upon CO loss following photoactivation, the Mn(I) centre is oxidized to Mn(II), possibly mononuclear, and to dinuclear Mn(III)Mn(III) and Mn(III)Mn(IV) species [41, 42] while the tpa ligand remains coordinated to the metal. Liposomes illuminated for 6 min without the PhotoCORM or with PhotoCORM in the absence of liposomes resulted in no changes in fluorescence (data not shown).

To further investigate membrane damage by [Mn(CO)_3_(tpa-κ^3^*N*)]Br, the membrane potential-sensitive dye diSC_3_(5) was used to determine the depolarizing effects of the PhotoCORM on the cytoplasmic membrane of *E. coli* EC958. Fluorescence caused by the release of diSC_3_(5) from the membrane in response to non-illuminated PhotoCORM was monitored over a period of 150 s (Fig. 3e). Dose-dependent increases in fluorescence were observed on the addition of the PhotoCORM up to a concentration of 200 µM. As described previously [25], the title compound elicits greater antibacterial activity when illuminated at 365 nm in the presence of bacterial cells. However, although the optimal excitation wavelength for diSC_3_(5) is 660 nm (emission 675 nm), some excitation occurred with our light source at 365 nm, so it was only necessary to photoactivate the CORM prior to addition of the diSC_3_(5). Illuminated PhotoCORM caused some increase in membrane depolarization at all of the tested concentrations (Fig. 3f), beyond that of the non-illuminated compound. Together, these results strongly suggest that the PhotoCORM disrupts membranes, explaining in part the synergy with other membrane-active compounds, such as polymyxins and the human peptide LL37.

### The tetracycline resistance gene *tetA* of EC958 is down-regulated by PhotoCORM and DOX

The EC958 genome includes pEC958 bearing the *tetA* gene [26] that encodes an energy-dependent tetracycline efflux pump conferring resistance to tetracyclines [43]. As predicted, *tetA* was up-regulated in response to DOX (by over 40-fold), in accordance with the resistance of EC958 to DOX (Table S3). However, when EC958 cells were treated with a combination of DOX and PhotoCORM, *tetA* up-regulation was substantially lower (only 18-fold), suggesting that the PhotoCORM may impair the efflux of DOX, thus potentiating its activity.

### Evidence for DNA damage by PhotoCORM in combination with DOX or CST

To seek other damaging effects of [Mn(CO)_3_(tpa-κ^3^*N*)]Br in combination with CST or DOX we measured the expression of *recA*. Bacteria respond to DNA damage by inducing genes for DNA repair and control of cell division. This forms part of the SOS response and is regulated by the RecA protein [44]. Treatment of cells with the PhotoCORM and CST showed no changes in the expression of *recA* (not shown). However, in response to a combination of the PhotoCORM and DOX, *recA* was up-regulated by almost fourfold (Table S3), whereas neither compound alone significantly increased transcription (Table S3). Thus, increased DNA damage from a combination the two compounds may contribute to the observed synergy.

### The effects of PhotoCORM and antibiotics on genes responsive to reactive oxygen species (ROS)

Others have attributed the toxic effects of CORMs on bacteria to the generation of ROS [45, 46] but treatments of *E. coli* with CORM-3 [10], CO gas [47], the present PhotoCORM [25] or the manganese carbonyl complex CORM-401 (L K Wareham and R K Poole, submitted) do not significantly up-regulate the expression of genes involved in the response to ROS. We therefore investigated whether combining sub-inhibitory concentrations of [Mn(CO)_3_(tpa-κ^3^*N*)]Br with antibiotics altered the expression of genes responsive to ROS. The transcript levels of *sodA* (encoding superoxide dismutase) were unaltered following treatment with PhotoCORM (Table S3), whereas DOX alone led to a twofold down-regulation of *sodA* (Table S3). Notably, when cells were treated with a combination of PhotoCORM and DOX, *sodA* was down-regulated fourfold (Table S3). Other ROS-responsive genes such as *ahpC,* which encodes alkyl hydroperoxide reductase, the primary scavenger of endogenous hydrogen peroxide in *E. coli*, and *katG*, a H_2_O_2_-detoxifying catalase, showed no significant changes in gene expression in response to treatment with PhotoCORM +/-DOX or CST (data not shown). Collectively, these data do not support the view that the PhotoCORM elicits oxidative stress; down-regulation of *sodA* and consequential sensitivity to ROS may contribute to the synergistic effects of DOX and PhotoCORM.

### Activated PhotoCORM chelates iron and induces production of iron-chelating substances by *E. coli* EC958

**Fig. 4.**
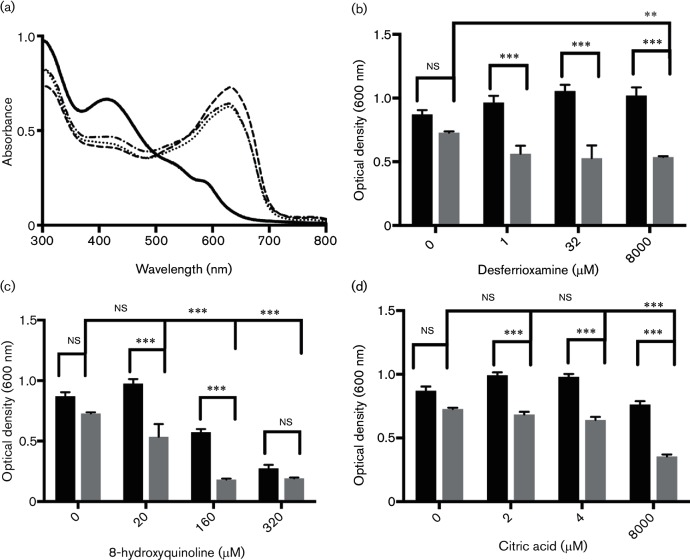
[Mn(CO)_3_(tpa-κ^3^*N*)]Br exacerbates growth inhibition in iron-restricted medium and triggers production of siderophore-like activity. (a) Culture supernatants were treated either with (solid line) or without (dot-dashed line) the PhotoCORM (300 µM) before pre-illumination at 365 nm for 6 min. Additional controls are the PhotoCORM (300 µM) illuminated in Tris-HCl buffer (dotted line) and Tris-HCl buffer only (dashed line). Samples were treated with CAS assay solution and the absorbance spectra recorded; decreased A_630 nm_ indicates iron loss from CAS due to chelation by the sample. (b)–(d) Culture aliquots were shaken in a 96-well plate and supplemented with: (b) desferrioxamine; (c) 8-hydroxyquinoline; and (d) citric acid. The graphs show the OD_600_ at 24 h. Black bars represent no PhotoCORM and the grey bars represent the addition of activated PhotoCORM (150 µM). The bars are the mean±SEM of three biological repeats. *, *P*<0.05; **, *P*<0.01; ***, *P*<0.001 (two-way ANOVA).

Treatment of EC958 with activated [Mn(CO)_3_(tpa-κ^3^*N*)]Br results in up-regulation of *entE*, which encodes an enzyme involved in enterobactin biosynthesis; exposure of anaerobically grown *E. coli* cultures to CO gas also perturbs iron levels [25, 47]. This led us to hypothesize that the title compound might trigger an iron starvation response in strain EC958. To establish whether the transcript levels of iron acquisition genes were reflected in cell physiology, we performed chrome Azurol S (CAS) assays [39] for siderophore activity. The CAS dye is blue when bound to Fe(III) and becomes orange (decreased absorbance at 630 nm) when the iron reacts with a ligand of higher affinity. Cells were grown to the late exponential phase (OD_600_~0.8–1.0), suspended in Tris-HCl buffer and then treated with the PhotoCORM or CO-depleted PhotoCORM (both at 300 µM). where appropriate, before illumination. The supernatants from these samples were then incubated with CAS assay solution for 1 h at room temperature, and the spectra were recorded. Culture supernatants from bacteria that were treated with the PhotoCORM and then illuminated (Fig. 4a, solid line) showed a significantly lower A_630_ value compared to that of the Tris buffer control (*P*<0.0001, Fig. 4a, dashed line), whereas the supernatant from control cultures not treated with the PhotoCORM (Fig. 4a, dot-dashed line) and the PhotoCORM pre-illuminated in buffer (Fig. 4a, dotted line) did not change. One explanation for these results is that activated PhotoCORM induces the secretion of siderophores or other high-affinity Fe(III) ligands; another is that activated PhotoCORM, or a subsequent breakdown product [41, 42], directly chelates iron. [Mn(CO)_3_(tpa-κ^3^*N*)]Br is unlikely to bind iron because the manganese tricarbonyl moiety occupies the binding pocket of the tpa co-ligand; however, upon illumination and CO loss, the manganese–tpa fragment or the liberated tpa ligand may bind iron. Alternatively, manganese may displace iron on the dye.

To explore these possibilities, we investigated whether the free tpa ligand or a simple Mn(II) salt, manganese(II) perchlorate, decreased absorbance in the CAS assay. The Mn(II) salt (300 µM) did not reduce A_630_, either following illumination or when kept dark (not shown), but high concentrations of tpa (1.5 mM) in the presence of cells did reduce A_630_ compared to that of the supernatant alone (Table 2, lines 5, 8), although the difference was not significant (*P*>0.05). In summary, although cells grown with PhotoCORM show evidence of iron depletion, the strong iron-chelating capacity of supernatants from cells treated with the illuminated PhotoCORM is caused in part by iron chelation by the tpa ligand and also by compounds such as siderophores that are produced by EC958 in response to the activated PhotoCORM. Interestingly, like [Mn(CO)_3_(tpa-κ^3^*N*)]Br, the tpa ligand caused a larger decrease in absorbance at 630 nm when added in the presence of cells compared to when in buffer alone (Table 2, lines 5 and 9; *P*<0.05), suggesting that tpa is also able to trigger the production of iron-chelating substances in cultures of EC958. Only a slight decrease in A_630_ was observed for cultures treated with higher concentrations of the PhotoCORM (1.5 mM) in Tris buffer kept in the dark (data not shown), providing further evidence that the intact PhotoCORM does not chelate iron.

**Table 2.** Iron chelation assays Data are listed in order of decreasing iron-chelating activity (increasing A_630_). Samples of PhotoCORM (+=300 µM), DOX (+=9 µg ml^−1^, ++=96 µg ml^−1^), or the ligand tpa (+=300 µM, ++=1.5 mM) were added to either Tris-HCl buffer, 50 mM, pH 7.4, or to a suspension of EC958 in the same buffer. '–' indicates that this compound/condition was not present in this sample. Samples were kept in the dark unless stated, in which case they were illuminated *in situ* at 365 nm for 6 min. Samples containing bacteria were then centrifuged (4000 r.p.m.) and 500 µl supernatant was incubated at room temperature for 1 h with 500 µl CAS dye. A UV visible spectrum was then recorded for each sample (300–800 nm). The mean A_630_ value for each sample is shown here ±sd (*n*=3).

	PhotoCORM	tpa	DOX	Light *in situ*	EC958 cells	A_630_
1	+	−	−	+	+	0.08±0.01
2	+	−	+	+	+	0.17±0.11
3	−	−	++	−	+	0.30±0.02
4	−	−	++	−	−	0.35±0.07
5	−	++	−	−	+	0.42±0.07
6	−	+	−	−	+	0.55±0.03
7	−	−	+	−	+	0.56±0.02
8	−	−	−	−	+	0.58±0.11
9	−	++	−	−	−	0.59±0.03
10	−	−	+	−	−	0.63±0.03
11	+	−	−	−	−	0.65±0.06
12	−	+	−	−	−	0.71±0.04

### Iron chelation exacerbates the antimicrobial effects of PhotoCORM, while iron supplementation reduces antimicrobial activity

The ability of [Mn(CO)_3_(tpa-κ^3^*N*)]Br to trigger an iron starvation response suggested that iron chelators would exacerbate the antimicrobial activity of activated PhotoCORM. Bacteria were therefore grown with chelators with different stability constants for Fe(II) and Fe(III): desferrioxamine [log stability Fe(III)=30.6], citric acid [log stability Fe(II)=4.4 and Fe(III)=11.4], or 8-hydroxyquinoline [log stability Fe(II)=15 and Fe(III)=26.3] [48, 49]. While treatment of cells with the activated PhotoCORM alone (150 µM) did not cause significant reductions in cell growth compared to untreated cells (Fig. 4b–d), growth (OD at 600 nm) was significantly inhibited when it was combined with increasing concentrations of desferrioxamine (Fig. 4b), 8-hydroxyquinoline (Fig. 4c) or citric acid (Fig. 4d) (*P*≤0.001). To test the effect of the converse treatment, i.e. provision of additional iron, EC958 cultures were grown in either normal or Fe-supplemented media (10-fold or 50-fold increases in the basal Fe concentration of 31 µM) in the presence or absence of the activated [Mn(CO)_3_(tpa-κ^3^*N*)]Br (250 µM). Iron supplementation of the medium reduced the antimicrobial activity of the PhotoCORM (Fig. S3). Having shown that iron starvation is a major player in the antimicrobial activity of [Mn(CO)_3_(tpa-κ^3^*N*)]Br, we used ICP-MS to measure the intracellular iron levels in EC958 cells treated for 30 min with the activated PhotoCORM at a concentration causing substantial inhibition of growth [25] (300 µM); the intracellular iron levels were 60 % lower following treatment with the PhotoCORM compared to untreated cells (183 and 459 µM respectively), confirming the ability of the title compound to deplete intracellular iron.

### The role of iron in the combined antimicrobial activity of DOX and PhotoCORM

The suppression of bacterial growth by tetracyclines may in part be caused by iron limitation [50, 51], while high iron levels block the accumulation of tetracyclines [52]. Tetracyclines chelate iron and lower DOX concentrations are required to inhibit bacterial growth in low-iron environments [53]. We therefore investigated whether a combination of DOX and [Mn(CO)_3_(tpa-κ^3^*N*)]Br exacerbated iron chelation (Table 2). The addition of DOX (96 µg ml^−1^) to buffer significantly reduced the A_630_ of CAS compared with buffer alone (*P*<0.005), but a lower DOX concentration did not (Table 2, lines 4, 10). Interestingly, the addition of DOX to a culture elicited a greater reduction in A_630_ than its addition to buffer (*P*<0.05), although this difference was not significant at the lower concentration of DOX used (Table 2, lines 3 and 7). Thus, metal chelation by high concentrations of DOX may also cause the secretion of iron-chelating substances such as siderophores by strain EC958. When added to a bacterial cell suspension, the combination of DOX (9 µg ml^−1^) with activated PhotoCORM (300 µM) caused a greater decrease in A_630_ than DOX alone (Table 2, lines 2 and 7; *P*<0.05), but not compared to activated PhotoCORM alone (Table 2, line 1). Thus activated PhotoCORM in the presence of bacterial cells is sufficient to cause the maximum decrease in A_630_, and so is at the outer sensitivity limits of this assay. We conclude that [Mn(CO)_3_(tpa-κ^3^*N*)]Br, or rather its solution products, and DOX each inhibit bacterial growth by chelating iron, explaining in part why these compounds act synergistically.

**Fig. 5.**
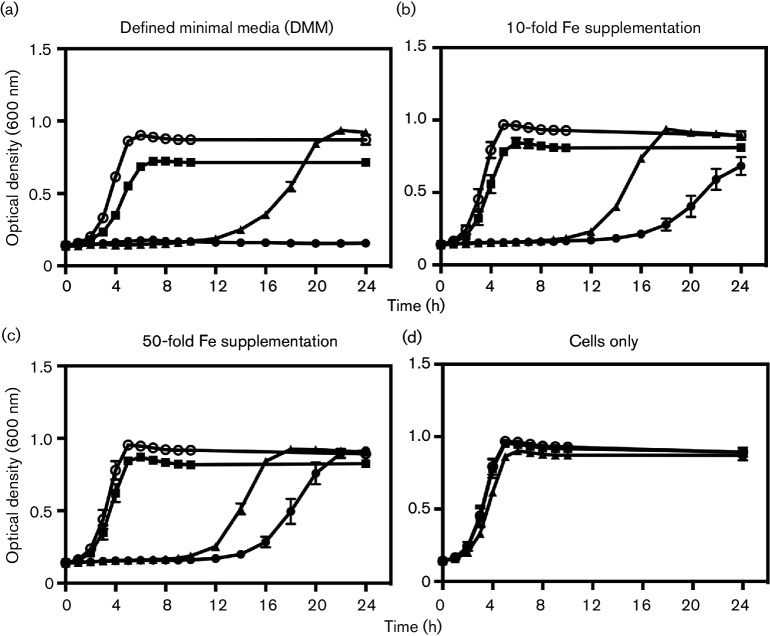
Iron supplementation reverses the combined toxicity of DOX and light-activated [Mn(CO)_3_(tpa-κ^3^*N*)]Br. Cells were grown in DMM (a), or media supplemented with 10-fold (b) or 50-fold (c) excesses of Fe(III). In (a), (b) and (c) are shown: cells in media alone (O), 200 µM activated PhotoCORM (■), 24 µg ml^−1^ DOX (▴) and activated PhotoCORM plus DOX (⚫). Controls with no additions (d) are: cells grown in DMM (▴), and 10-fold, and 50-fold (■) excesses of Fe(II). The bars are mean ±sem of three biological repeats.

The effect of Fe(III) supplementation on the synergistic effects of activated PhotoCORM and DOX was studied in bacterial cultures (Fig. 5). EC958 cells were grown in either minimal medium or medium supplemented with either a 10- or 50-fold excess of iron. Cells were treated with DOX (24 µg ml^−1^) and PhotoCORM (200 µM), which, in medium without Fe supplementation caused a prolonged and complete growth inhibition (Fig. 5a). However, in medium supplemented with 10-fold more Fe, sustained growth inhibition was not observed (Fig. 5b). This reversal of inhibition was more pronounced when 50-fold excess Fe was included in the medium (Fig. 5c). Cells started to recover at 14 h, reaching a similar OD_600_ to that of untreated cells at 24 h. Fig. 5dshows little difference in the growth of EC958 in minimal media with or without iron supplementation. Thus, iron chelation is critical in the observed synergy between the two antimicrobials.

### CO gas does not elicit iron-chelating activity and is unable to potentiate the antimicrobial activity of DOX in EC958

CO gas triggers an iron starvation response in anaerobic *E. coli* MG1655 cells, leading to the up-regulation of numerous iron acquisition genes and siderophore-like activity [47]. Since [Mn(CO)_3_(tpa-κ^3^*N*)]Br releases two CO groups per molecule upon illumination, we investigated the role of CO in siderophore production in aerobically grown EC958. The CAS assay was repeated, but cells were bubbled with CO gas (5 min) alone (A_630_=0.71±0.04) or in the presence of 300 µM tpa and Mn(ClO_4_)_2_ (A_630_=0.57±0.02), and the supernatant from these cultures was added to the CAS reagent. There was no significant difference (*P*>0.05) in A_630_ between samples treated with CO and the samples without CO treatment (A_630_=0.67±0.02), suggesting that CO does not contribute to the production of iron-chelating molecules in response to [Mn(CO)_3_(tpa-κ^3^*N*)]Br. When strain EC958 was treated with increasing concentrations of DOX (12–96 µg ml^−1^) and CO-saturated solution (400 µM), CO did not potentiate the antimicrobial activity of DOX (data not shown). The MIC of DOX with or without CO remained at 96 µg ml^−1^.

## Discussion

Bacterial infections endanger human health worldwide, a threat that is compounded by the relentless spread of antimicrobial resistance (AMR) and the slow development of new antibiotics. Observers claim that we are approaching the ‘post-antibiotic era’ [54]. Indeed, in 2015 researchers identified bacteria resistant to CST (the ‘drug of last resort’) in patients and livestock in China [55]. To meet these challenges, numerous international initiatives on AMR have been launched, including a World Health Organisation (WHO) global action plan. The spectre of global antibiotic resistance demands: (1) an accelerated search for new antibiotics via private/public partnerships; (2) better understanding of how non-antibiotic antimicrobial agents act and can therefore be used synergistically with established antibiotics [56]; (3) a search for new antimicrobials with distinct modes of action (e.g. CORMs) [16]; and (4) deeper understanding of how the innate immunity system can be used to aid clearing of bacterial infections.

The WHO recently announced a global priority list of antibiotic-resistant bacteria and identified antibiotic-resistant bacteria of the Enterobacteriaceae, including *E. coli,* as being amongst the most critical [57]. Therefore the search for novel treatment strategies is especially important for multi-drug resistant strains such as EC958 [26]. We had already briefly described the ability of [Mn(CO)_3_(tpa-κ^3^*N*)]Br to potentiate the antimicrobial effect of DOX [25], but the current work is the first comprehensive study of synergy between a range of antibiotics and a PhotoCORM. We show that [Mn(CO)_3_(tpa-κ^3^*N*)]Br potentiates the antibacterial activity of tetracycline and polymyxin antibiotics. Multifactorial effects are evident. First, the PhotoCORM disrupts membranes, leading to the reduced MIC of the combined treatments. The cationic polypeptides CST and PMB interact with anionic lipopolysaccharide (LPS) molecules in the outer membrane of Gram-negative bacteria and displace calcium and magnesium ions, thus disrupting the cell membrane [58, 59]. Although the observed synergies may therefore result from increased entry of the PhotoCORM into bacteria and enhanced access to intracellular targets, we found no increased intracellular accumulation of Mn (and thus the PhotoCORM) in the presence of antibiotics (Fig. S2). Second, the PhotoCORM or, more likely, the products of photoactivation, but not CO, bind iron and trigger an iron starvation response in strain EC958, including the production of siderophore-like activity. The observed synergy may reflect the iron-chelating activity of both the PhotoCORM and tetracyclines. Tetracyclines chelate iron [51–53] and antibiotics such as DOX and MIN are more effective in iron-restricted environments. The antimicrobial action of tetracyclines against *Plasmodium falciparum* infections is inhibited by iron and a catechol inhibitor, FR160, potentiates the activity of DOX [60]. Bacteria colonizing the urinary tract face extremely low iron availability; consequently, UPEC strains such as EC958 express a wide variety of iron acquisition systems, such as siderophore production, in order to survive inside the host [61]. Finally, reduced expression of the *tetA* gene encoding a tetracycline efflux pump may contribute to the synergy observed.

**Fig. 6.**
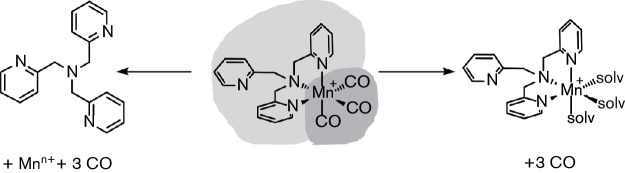
The ‘Janus-headed’ activity of [Mn(CO)_3_(tpa-κ^3^*N*)]Br. The title compound has multi-faceted properties, in which either the Mn(CO)_3_ moiety acts as a photolabile protective group for the tpa ligand (left) or the Mn(tpa) fragment acts as a carrier for carbon monoxide (right).

Although CO-releasing molecules (CORMs) have been heralded as promising and novel antimicrobial agents, there has been uncertainty about their mode(s) of action. However, the present results clearly demonstrate a lack of significant CO-mediated effects in the case of [Mn(CO)_3_(tpa-κ^3^*N*)]Br. CO-depleted PhotoCORM was able to potentiate the activity of CST to the same extent as the activated PhotoCORM. Similarly, cells treated with the non-illuminated PhotoCORM, i.e. with all CO ligands still attached, also showed some potentiation of DOX against EC958 cells. Cells treated with CO gas and DOX, on the other hand, showed no combined effect. These findings are in agreement with the study by Wareham *et al*. [47], which showed that, in contrast to the protective effects of other gasotransmitters such as NO and H_2_S [62, 63], CO gas had no effects, neither protection nor potentiation, against several classes of antibiotics [47]. This suggests a ‘Janus-headed’ activity of the title compound, where the Mn(tpa) fragment may act as a carrier for up to three CO ligands (with subsequent CO binding to terminal oxidases and respiratory inhibition [20, 25]), whereas the carbonyl ligands also act as photolabile ‘protective groups’, which prevent the biological activity of the Mn(tpa) fragment while coordinated (Fig. 6).

Nevertheless, CORMs have shown promise in combination therapy in a number of bacterial species. Sub-lethal doses of CORM-2 were combined with metronidazole, amoxicillin or clarithromycin and were found to potentiate antibiotic effects on clinical isolates of *H. pylori* [64]. Similarly, CORM-2 was found to act as an adjuvant to tobramycin against *P. aeruginosa* biofilms [65]. CORM-2 potentiates the effects of DOX, CTX and TMP [47]. This study shows for the first time that the PhotoCORM [Mn(CO)_3_(tpa-κ^3^N)]Br potentiates the activities of several antibiotics, and suggests future strategies to enhance antimicrobial activity against such multi-drug-resistant pathogens.

## Supplementary Data

311 Supplementary File 1
