# Supplementary material for: A manganese photosensitive tricarbonyl molecule [Mn(CO)3(tpa-κ3N)]Br enhances antibiotic efficacy in a multi-drug-resistant Escherichia coli
Source: Microbiology (Reading). 2017 Sep 28;163(10):1477–89. doi: 10.1099/mic.0.000526 (PMC5845575; doi:10.1099/mic.0.000526)
Supplement: Supplementary File 1 [file mic-163-1477-s001.pdf]

Supplementary Material

A Manganese Photoactivated Carbon Monoxide-Releasing Molecule $[Mn(CO)_3(tpa\text{-}\kappa^3N)]Br$ enhances antibiotic efficacy in a multi-drug resistant *Escherichia coli*

Namrata Rana[a], Helen E. Jesse[a], Mariana Tinajero-Trejo[a*], Jonathan A. Butler[a**], John D. Tarlit[a], Milena L. von und zur Mühlen[a], Christoph Nagel[b], Ulrich Schatzschneider[b] and Robert K. Poole[a#]

Department of Molecular Biology and Biotechnology, The University of Sheffield, Sheffield, United Kingdom[a] and the Institut für Anorganische Chemie, Julius-Maximilians-Universität Würzburg, Germany[b]

Contents: Table S1, S2, S3 and Fig. S1, S2, S3

Table S1: Comparison of antibiotic sensitivities of *E. coli* MG1655 and EC958 grown on Mueller Hinton II agar.

	MG16155		EC958	
**Antibiotics**	**MIC (µg/ml)**	**R/S[1]**	**MIC (µg/ml)**	**R/S**
Colistin (CST)	0.38	S	0.50	S
Doxycycline (DOX)	2.0	S	12	R
Gentamicin (GEN)	0.38	S	1.5	S
Kanamycin (KAN)	0.38	S	*	R
Minocycline (MIN)	3	S	3	S
Tetracycline (TET)	1	S	48	R
Trimethoprim (TMP)	0.25	S	*	R

[1]R and S stand for resistance and sensitivity to antibiotics respectively. *No zones of inhibition were observed and MICs could not be determined.

Table S2: Calculation of Fractional Inhibitory Concentrations (FICs) from checkerboard experiments.

Bacterial Strain	Antibiotic	$MIC_{PhotoCORM}$ alone (mM)	$MIC_{PhotoCORM}$ with Antibiotic (mM)	$FIC_{PhotoCORM}$*	$FIC_{Antibiotic}$ †	ΣFIC
EC958	CST	4.00	1.00	0.250	0.125	0.375
	DOX	4.00	0.125	0.031	0.250	0.281
	LL37	4.00	1.00	0.250	0.250	0.5
	OTC	4.00	4.00	1.00	-	>1.00
	PMB	4.00	0.500	0.125	0.125	0.25
	TET	4.00	2.00	0.500	0.250	0.75
MG1655	OTC	4.00	1.00	0.250	0.0625	0.313
	TET	4.00	0.250	0.0625	0.250	0.313

* $FIC_{PhotoCORM}$ = $MIC_{PhotoCORM}$ alone/$MIC_{PhotoCORM}$ with antibiotic. † $FIC_{Antibiotic}$ calculated from values shown in Table 1, i.e. $FIC_{Antibiotic}$ = $MIC_{Antibiotic}$ alone/$MIC_{Antibiotic}$ with PhotoCORM.

TABLE S3: Levels of gene expression assessed by RT-PCR in response to the PhotoCORM alone or in combination with DOX

Sample condition	Genes involved in:		
	Resistance to tetracyclines *tetA*	Response to DNA damage *recA*	Responses to reactive oxygen species *sodA*
PhotoCORM	0.77 ± 0.100	1.45 ± 0.072	0.97 ± 0.039
PhotoCORM+ DOX	18.2 ± 1.28	3.62 ± 0.265	0.26 ± 0.066
DOX	42.9 ± 6.06	1.43 ± 0.229	0.51 ± 0.211

Cultures of EC958 were treated with PhotoCORM (150 μM), alone or in combination with DOX (9 μg/ml). A culture was also treated with DOX alone as a control. The expression of individual genes of interest relative to the housekeeping gene *gyrA* was then compared with untreated cells (n = 3 ± S.E.M). A value of 1 indicates no change in gene expression relative to untreated cells, values >1 indicate up-regulation and values <1 indicate down-regulation. For example, a 2- fold increase is given as 2, and a 2-fold decrease is given as 0.5.

[Drug A: PhotoCORM] (columns); **[Drug B: Antibiotic]** (rows)

	MIC / 8	MIC /4	MIC /2	**MIC**	2 X MIC	4 X MIC	8 X MIC
MIC / 8							
MIC / 4							
MIC / 2							
**MIC**							
2 X MIC							
4 X MIC							
8 X MIC							

$$FIC_{PhotoCORM} = \frac{MIC_{PhotoCORM} \text{ in combination}}{MIC_{PhotoCORM} \text{ alone}}$$

$$FIC_{antibiotic} = \frac{MIC_{antibiotic} \text{ in combination}}{MIC_{antibiotic} \text{ alone}}$$

$$\Sigma FIC = FIC_{PhotoCORM} + FIC_{Antibiotic}$$

Fig. S1 Checkerboard analysis used to determine interaction of the title compound with antibiotics. The figure shows a typical setup of each checkerboard experiment. Increasing concentrations of PhotoCORM were administered to cells along the abscissa and increasing concentrations of antibiotic along the ordinate in a 96 well plate. The resulting checkerboard contains each combination of the two compounds, with wells that contain the highest concentration of each compound at opposite corners. The plate was incubated for 24 h at 37°C with shaking. From the resulting growth curves for each well, the MIC of each compound alone or in combination were determined. This was used to calculate the Fractional Inhibitory Concentration (FIC) for the two compounds. The ΣFIC is then used to determine the type of interaction between the two compounds. The combination is considered synergistic when the ΣFIC is ≤ 0.5, non-interacting when > 0.5 and < 4 and antagonistic when the ΣFIC is ≥ 4.

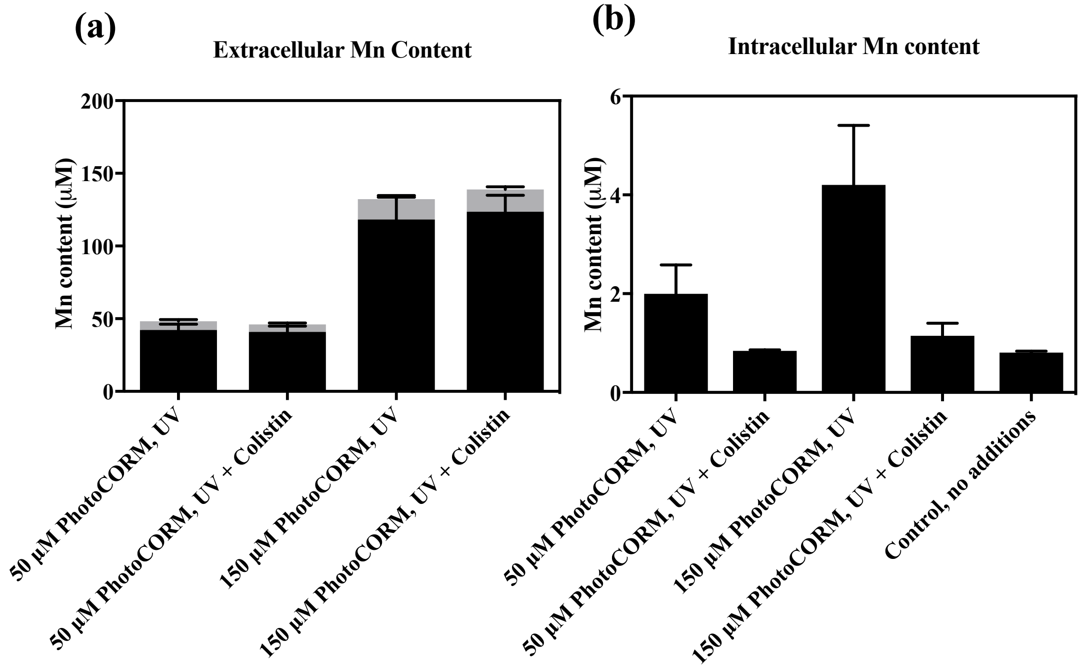

Fig. S2 Metal analyses by ICP-MS of cells treated with a combination of [Mn(CO)$_3$(tpa-κ^3*N*)]Br, UV and CST. Cultures were grown with either 50 µM or 150 µM UV-activated PhotoCORM, or combined with CST (0.5 µg/ml). In (a), extracellular Mn was quantified from the supernatants (black bars) and washes of cell pellets to remove loosely bound Mn (grey bars) after exposure to the PhotoCORM with or without CST. In (b), intracellular Mn was quantified in harvested cells on incubation with either the PhotoCORM alone or in combination with CST. Bars are mean ± SEM of three biological repeats.

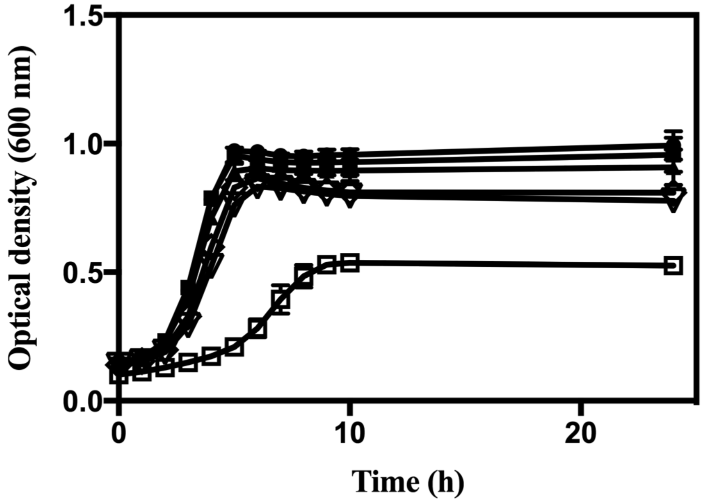

Fig. S3 Iron supplementation of growth media reduces the toxicity of light-activated $[Mn(CO)_3(tpa\text{-}\kappa^3 N)]Br$. Cells were grown in DMM (▲), or DMM supplemented with 10-fold (●), or 50-fold (■) excesses of Fe(III). Cells were treated with light-activated 250 μM PhotoCORM in DMM (□) or DMM supplemented with 10-fold (▽) or 50-fold (◇) excesses of Fe (III). Bars are mean ± SEM of three biological repeats.
